# Application of a recombinase polymerase amplification (RPA) assay and pilot field testing for *Giardia duodenalis* at Lake Albert, Uganda

**DOI:** 10.1186/s13071-020-04168-1

**Published:** 2020-06-06

**Authors:** Sandra J. Molina-Gonzalez, Tapan Bhattacharyya, Hajri R. AlShehri, Kate Poulton, Stephen Allen, Michael A. Miles, Moses Arianitwe, Edridah M. Tukahebwa, Bonnie Webster, J. Russell Stothard, Amaya L. Bustinduy

**Affiliations:** 1 Department of Clinical Research, London School of Hygiene & Tropical Medicine, London, UK; 2 London Centre for Neglected Tropical Disease Research, London, UK; 3 Department of Infection Biology, London School of Hygiene & Tropical Medicine, London, UK; 4 Department of Parasitology, Liverpool School of Tropical Medicine, Liverpool, UK; 5 Ministry of Health, Asir District, Abha, Kingdom of Saudi Arabia; 6 Natural History Museum Parasites and Vectors Division, Life Sciences Department, London, UK; 7 Department of Clinical Sciences, Liverpool School of Tropical Medicine, Liverpool, UK; 8 Vector Control Division, Ministry of Health, Kampala, Uganda

**Keywords:** *Giardia duodenalis*, *Giardia intestinalis*, *Giardia lamblia*, Point-of-care, Recombinase polymerase amplification, Giardiasis, Assemblage typing, Uganda, Epidemiology

## Abstract

**Background:**

*Giardia duodenalis* is a gastrointestinal protozoan causing 184 million cases of giardiasis worldwide annually. Detection is by microscopy or coproantigen assays, although sensitivity is often compromised by intermittent shedding of cysts or trophozoites, or operator expertise. Therefore, for enhanced surveillance field-applicable, point-of-care (POC), molecular assays are needed. Our aims were to: (i) optimise the recombinase polymerase amplification (RPA) assay for the isothermal amplification of the *G. duodenalis* β-giardin gene from trophozoites and cysts, using published primer and probes; and (ii) perform a pilot field validation of RPA at a field station in a resource-poor setting, on DNA extracted from stool samples from schoolchildren in villages around Lake Albert, Uganda. Results were compared to an established laboratory small subunit ribosomal RNA (*SSU* rDNA) qPCR assay with additional testing using a qPCR targeting the triose phosphate isomerase (*tpi*) DNA regions that can distinguish *G. duodenalis* of two different assemblages (A and B), which are human-specific.

**Results:**

Initial optimisation resulted in the successful amplification of predicted RPA products from *G. duodenalis*-purified gDNA, producing a double-labelled amplicon detected using lateral flow strips. In the field setting, of 129 stool samples, 49 (37.9%) were positive using the *Giardia/Cryptosporidium* QuikChek coproantigen test; however, the RPA assay when conducted in the field was positive for a single stool sample. Subsequent molecular screening in the laboratory on a subset (*n* = 73) of the samples demonstrated better results with 21 (28.8%) RPA positive. The *SSU* rDNA qPCR assay resulted in 30/129 (23.3%) positive samples; 18 out of 73 (24.7%) were assemblage typed (9 assemblage A; 5 assemblage B; and 4 mixed A+B). Compared with the *SSU* rDNA qPCR, QuikChek was more sensitive than RPA (85.7 *vs* 61.9%), but with similar specificities (80.8 *vs* 84.6%). In comparison to QuikChek, RPA had 46.4% sensitivity and 82.2% specificity.

**Conclusions:**

To the best of our knowledge, this is the first in-field and comparative laboratory validation of RPA for giardiasis in low resource settings. Further refinement and technology transfer, specifically in relation to stool sample preparation, will be needed to implement this assay in the field, which could assist better detection of asymptomatic *Giardia* infections.
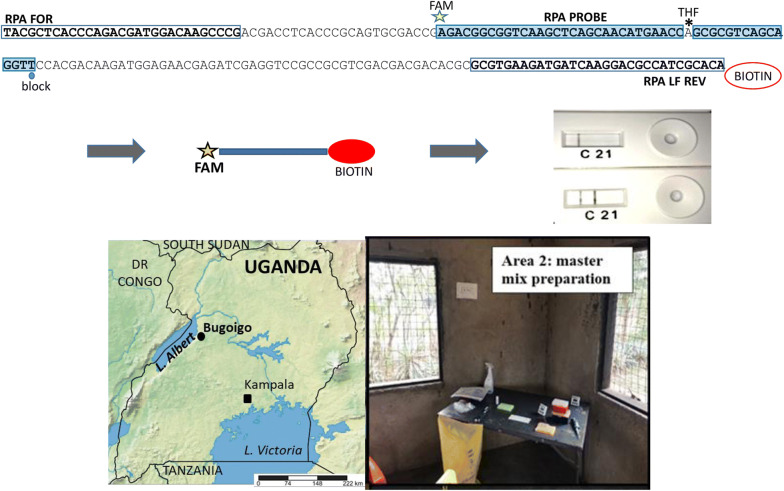

## Background

*Giardia duodenalis* (syns *G. lamblia* and *G. intestinalis*) is a flagellated protozoan parasite of the human gastrointestinal tract as well as a range of other mammals worldwide. It is acquired by ingestion of viable cysts present in faecal-contaminated water, food or on fomites [1]. Ingested cysts undergo excystation into actively multiplying trophozoites in the duodenum, which attach to the intestine causing epithelial inflammation, villous flattening and malabsorption. Symptoms include acute or chronic diarrhoea, weight loss and impaired development in children [1, 2]. The encysted stage is passed in stools [2]. Symptomatic giardiasis results in around 184 million clinical cases globally per year. *Giardia duodenalis* infection can be asymptomatic and undetected carriers remain a source of infection. On the basis of molecular characterisation, *G. duodenalis* is divided into eight genetic assemblages (A–H), of which A and B are considered human-specific [3]; thus, it is important to identify which assemblages are present during validation of any new molecular diagnostic based on species-specific DNA loci.

Detection of *G. duodenalis* is routinely carried out by identifying cysts or trophozoites in faeces using direct microscopy, with variable sensitivity and specificity. These methods can be labour-intensive, requiring multiple examinations or involve complicated concentration procedures best performed by experienced technicians [4, 5]. Immunological and molecular techniques have achieved better sensitivity of 79–100% [6] but specificity can be reduced. A drawback of molecular assays is often the need for expensive and sophisticated equipment that is not available in resource-poor endemic settings. Giardiasis is particularly common in Uganda [7]. Al-Shehri et al. [8] reported a prevalence of *G. duodenalis* infection at 42% (40/96) using the rapid QuikChek coproantigen test and 87% (221/254) by qPCR in Ugandan children (5–10 years-old), with many heavy infections.

Unlike conventional PCR-based methods, recombinase polymerase amplification (RPA) is an isothermal amplification system that is rapid and requires only basic and portable equipment [9] making it feasible for the point-of-care (POC) diagnosis of tropical diseases in low resource endemic settings. Crannell et al. [10] developed an RPA assay for *G. duodenalis* amplifying a fragment of the β-giardin gene. Here, we further optimised this RPA assay for *G. duodenalis* for use in a resource-poor setting, and tested its applicability at a field station in a remote rural area near Lake Albert, Uganda, highly endemic for giardiasis. Additionally, we utilised the commercially available *Giardia*/*Cryptosporidium* QuikChek coproantigen test (Abbott, Maidenhead, UK), in the field to investigate the prevalence of giardiasis in a cohort of children from the endemic area of Lake Albert. Samples from this cohort were also analysed using the small subunit ribosomal RNA gene (*SSU* rDNA) qPCR assay for *Giardia* and also the triose phosphate isomerase (*tpi*) qPCR assay to enable discrimination of assemblage A and B *G. duodenalis* [11]. RPA and the QuikChek coproantigen test were compared against the *SSU* rDNA qPCR as the ‘gold standard’.

## Methods

### Laboratory RPA optimisation

#### Giardia duodenalis genomic DNA controls

Cryopreserved *G. duodenalis* trophozoite pellets, assemblage A (sourced from the London School Hygiene and Tropical Medicine (LSHTM), London, UK) and *G. duodenalis* cysts H-3 (human isolate, assemblage B; P101, Waterborne Inc., New Orleans, USA) were used as sources of control DNA for the testing of the RPA and qPCR assays. The control DNA samples were extracted using the QIAamp DNA Mini Kit (51304; Qiagen, Manchester, UK), with modifications: cells were mixed with 1 ml of NucliSENS lysis buffer (supplied as containing 50% guanidine thiocyanate, < 2% Triton X-100, < 1% EDTA) (200292; BioMerieux, Basingstoke, UK) and Precellys Soil Mix beads SK38 (03961-1-006; Stretton Scientific, Stretton, UK). Samples were vortexed for 5 min, and incubated at room temperature for 20 min. Beads and debris were pelleted by centrifugation at 14,000× *rpm* for 2 min. Two hundred microliters of supernatant was transferred to a clean microcentrifuge tube and the manufacturer’s protocol for the Qiagen 51304 QIAamp DNA Mini Kit was followed thereafter, with the DNA being eluted in 200 µl of AE buffer (10 mM Tris-Cl, 0.5 mM EDTA; pH 9.0).

#### Recombinase polymerase amplification (RPA) and lateral flow G. duodenalis assay

**Fig. 1:**
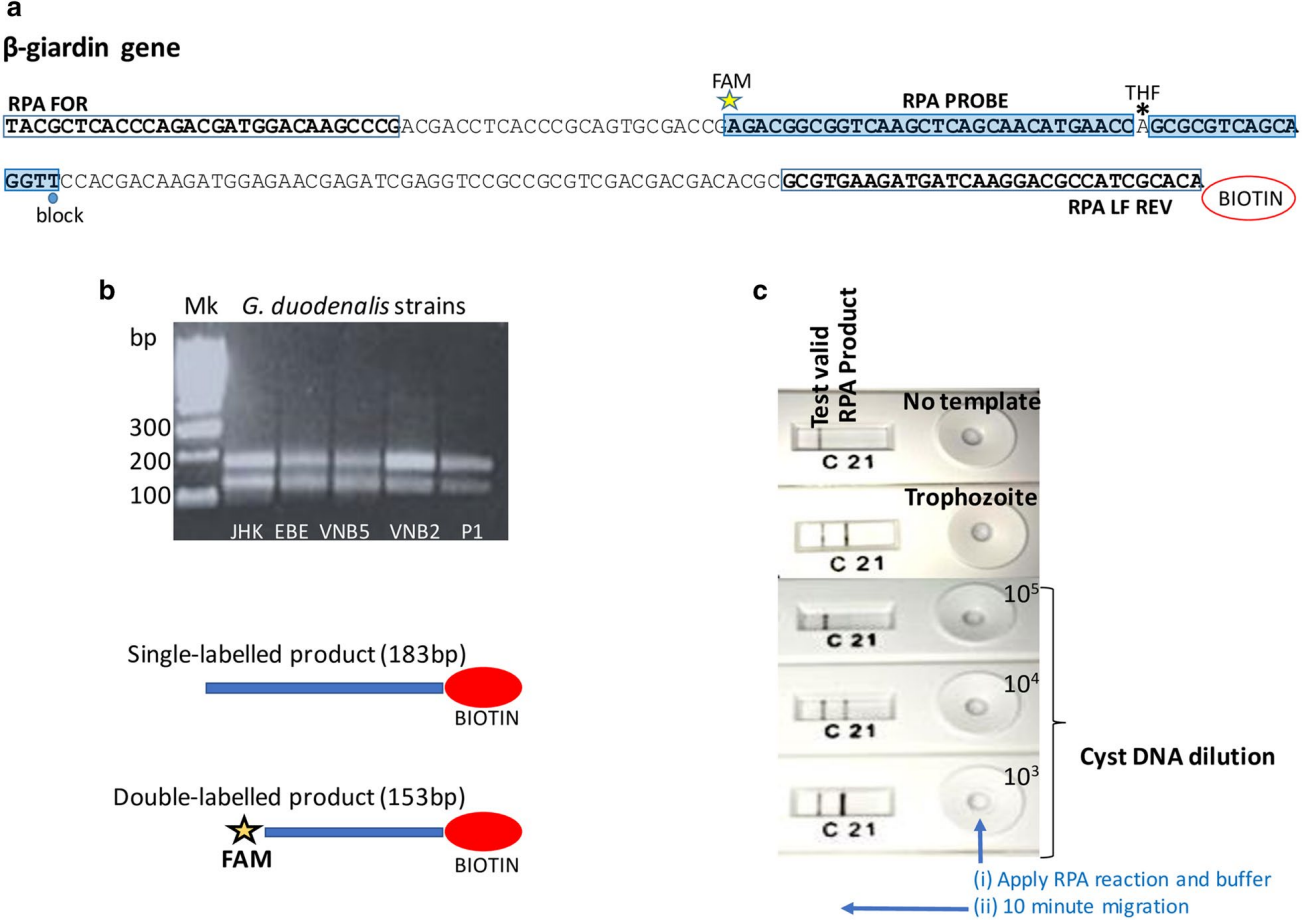
RPA of the *G. duodenalis* β-giardin gene. **a** RPA primers amplify nts 796–978 of GenBank X85958: binding sites of primers (clear boxes) and probe (shaded box) with modifications of the probe (5’ FAM, internal THF, 3’ block) and the reverse primer (5’ biotin); the reverse primer is depicted on the corresponding sequence of the sense strand. **b** Identification of predicted amplification products by gel electrophoresis. See Methods for the derivation of the two amplicons. **c** Presence of the double-labelled RPA product detected visually on the lateral-flow cassette from trophozoite and cyst DNA. *Abbreviations*: Mk, DNA size marker; nts, nucleotides; THF, tetrahydrofuran

All primer and probe sequences used were as previously described [10] (Additional file 1: Table S1). Figure 1a depicts the binding locations on the RPA probes and primers for the β-giardin gene.

Three different areas in the laboratory were assigned for (i) the RPA master mix preparation, (ii) the addition of DNA, and (iii) the RPA reaction and lateral flow amplicon detection in a laminar flow hood. This was done to prevent cross-contamination and false positives. Reactions were performed using the TwistAmp nfo kit (TANFO02KIT; TwistDx, Maidenhead, UK) in a final volume of 50 µl. The master mix consisted of 29.5 µl of rehydration buffer, 2.1 µl of 10 µM forward primer, 2.1 µl of 10 µM reverse primer, 0.6 µl of 10 µM probe, 7.5 µl of 5 M betaine (720 mM) (B0300; Sigma-Aldrich, Gillingham, UK) and 2.5 µl of H_2_O. The RPA nfo pellets were transferred to sterile individually capped 0.2 ml tubes, to which 37.5 µl of master mix was added. Then, 2.5 µl of 280 mM magnesium acetate was added to the inside wall of each tube. Ten microlitres of extracted DNA was added to the tubes, which were then inverted manually 8 to 10 times and centrifuged briefly. Negative controls (no template) were incorporated into each set of reactions.

Reactions were incubated at 37 °C for 30 min in a thermal cycler (Applied Biosystems 9700). At minutes 4 and 15 of the incubation, the tubes were removed from the cycler, inverted manually 8 to 10 times and then centrifuged briefly before being returned to 37 °C. After 30 min of incubation, the reactions were immediately placed at 4 °C. Rapid detection of the FAM-biotin double-labelled amplification product was visualised using lateral flow Milenia Genline HybriDetect (MGHD) strips in a dipstick format and also the PCRD Nucleic Acid Detector lateral flow cassettes (Abingdon Health, York, UK). Detections using the MGHD strips were performed by adding 2 µl of the RPA reaction to 98 µl of supplied detection buffer, into which the strips were then placed; after 5 min of incubation at room temperature the results were assessed visually as positive or negative by the presence or absence of the test line. For the PCRD cassettes, the following manufacturer’s recommendations were used: 5 µl of the RPA reaction was added to 70 µl of the cassette running buffer; the whole 75 µl was then added to the PCRD cassette window and incubated at room temperature for 10 min. These assays were also assessed visually by the presence or absence of the test line.

Successful RPA reactions were also assessed by electrophoresis of the reaction products. Amplicons were column-purified (28104: Qiagen) and then run on a 2% agarose gel to check for specific amplification of the 153 bp amplicon, i.e. the double-labelled RPA product.

### Field assessment at Lake Albert, Uganda

#### Stool collection

Single stool samples were collected from primary school children in three Ugandan villages on the shore of Lake Albert. Participant children were randomly selected from schools where studies of intestinal schistosomiasis were ongoing [12]. A field station laboratory was set up nearby at the Bugoigo research camp consisting of a small building with separate areas for DNA extraction from the stool samples, RPA preparation and amplicon detection. The field station was equipped with all the necessary resources for performing the stool DNA extractions and the RPA with electricity supplied by means of a portable generator. An aliquot of fresh stool (*c.*500 µl) was homogenized with PBS (1:1). A second aliquot of stool was prepared by homogenizing 500 µl of fresh faeces with 500 µl of 70% ethanol, in a 2.0 ml screw-cap tube; these aliquots were stored for later testing as described below.

#### Coproantigen test

An aliquot of stool was also tested at the field station with the commercially available *Giardia*/*Cryptosporidium* QuikChek coproantigen test (Abbott), according to manufacturer’s instructions. Briefly, the stool sample was added to the supplied diluent/conjugate mixture, transferred to the test cassette containing anti-*Giardia* monoclonal antibodies, and after addition of the supplied wash buffer and substrate, results were read visually.

#### Field stool DNA extraction for RPA

DNA was extracted from each stool sample homogenized in PBS using the QIAamp DNA Mini Kit (51304; Qiagen). Approximately 200 mg of the stool samples were transferred to a 2 ml screw-cap tube containing 1 ml of the NucliSENS lysis buffer and Precellys Soil Mix beads SK38. Samples were vortexed for 5 min, and incubated at room temperature for 20 min. Beads and debris were collected in a pellet by centrifugation at 14,000× *rpm* for 2 min. Two hundred microliters of the supernatant was transferred to a clean microcentrifuge tube and the manufacturer’s protocol was followed thereafter. DNA was eluted in 200 µl of AE buffer.

#### Field RPA analysis

RPA assays were performed in the field using the assay conditions as described above, except that 0.7 µl of 5 M betaine (70 mM) was added for each reaction. Amplification was carried out at 37 °C using a dry block heater (SP2280; SciQuip, Wem, UK). Detection of amplicons was performed using PCRD Nucleic Acid Detector lateral flow cassettes as described above.

### Subsequent laboratory testing of the field samples at LSHTM

#### Stool DNA extraction

Stool samples that had been stored in ethanol were washed 3 times with PBS and then extracted using the QIAamp DNA Stool Mini Kit (51504; Qiagen) following the manufacturer’s protocol, and eluted in 200 µl of supplied buffer AE. The DNA concentration was determined using a Nanodrop ND-1000 spectrophotometer (Thermo Fisher Scientific, Paisley, UK). The use of this kit, an alternative to the QIAamp DNA Mini Kit used in the original *Giardia* RPA assay published by Crannell et al. [10] and in this work at the Lake Albert field station, was employed here to remove potential inhibitors from the samples for downstream molecular assays. These assays were: RPA (as detailed above); assemblage-specific qPCR (*tpi* gene); and non-assemblage specific *SSU* qPCR test.

#### Assemblage-specific qPCR assays

The assemblage-specific multiplex qPCR [11] was performed on individual samples. Primers and probes are listed in Additional file 1: Table S1 and binding sites are shown in Additional file 2: Figure S1. Each reaction was performed in a total volume of 20 µl, and comprised 10 µl of 2× reaction mix (B20.23-01; PCR Biosystems, London, UK), 0.8 µl of a 10 µM solution of each forward and reverse primer for assemblage A and B, 0.4 µl of a 10 µM solution of each assemblage (A and B) probe, 4.4 µl of DNA, and 1.6 µl of ddH_2_O. Samples were run on a Rotor-Gene 3000 machine (Corbett Research, Mortlake, Australia) using the following cycling conditions: 1 cycle at 95 °C for 2 mins; 40 cycles at 95 °C for 5 s and 60 °C for 30 s. Each qPCR run included positive controls containing DNA extracted from trophozoite (assemblage A) and cyst (assemblage B). Thresholds to calculate Cq values were set compared to water controls in each run. Negative (no template) reactions were run with each set of reactions.

#### SSU rDNA qPCR

The *SSU* rDNA qPCR was performed on the same DNA extracts as described above using the primer and probe sequences as previously described [13, 14]. The primers and probe are listed in Additional file:1: Table S1 and binding sites are shown in Additional file 2: Figure S1. Reactions were performed in a total volume of 20 μl, and comprised 10 µl of 2× reaction mix (B20.23-01; PCR Biosystems), 0.8 µl of a 10 µM solution of each primer, 0.4 µl of a 10 µM solution of probe, 5 µl of DNA, and 3 µl of ddH_2_O. Samples were run on a Rotor-Gene 3000 as described above.

### Statistical tests

Confidence intervals (95% CI) for sensitivity and specificity comparisons of *SSU* rDNA qPCR, RPA and QuikChek were calculated using Graphpad QuickCalcs (https://www.graphpad.com/quickcalcs/ConfInterval1.cfm).

Receiver operating characteristic (ROC) curves for comparing RPA and the QuikChek coproantigen test against the *SSU* rDNA qPCR as the ‘gold standard’ were calculated using easyRoc v1.3.1 (http://www.biosoft.hacettepe.edu.tr/easyROC/). *P*-values of < 0.05 were considered significant.

## Results

### RPA optimisation at LSHTM

As shown in Fig. 1b, the RPA with DNA extracted from the *G. duodenalis* cultures generated amplicons of the predicted sizes from the β-giardin gene. The 153-bp FAM-biotin double-labelled product could be reliably identified by the PCRD lateral flow cassette; cyst DNA diluted to 10^4^ (approximately 6 ng/µl) generated RPA products detectable by lateral flow and confirmed by gel electrophoresis (Fig. 1c). As predicted from the binding sites of the RPA primers and probe (Fig. 1a), two amplicons were produced: the 153-bp double-labelled amplicon produced by the RPA probe with primer RPA LF REV; and the 183-bp amplicon produced by primers RPA FOR and RPA LF REV. As described by Piepenburg et al. [9] the RPA probe binds the 183-bp amplicon, allowing nfo endonuclease to expose a 3’-OH, and Bsu polymerase to generate the 153-bp double-labelled amplicon. We observed, during our initial optimisation phase that the MGHD strips were prone to producing false-positive results from water (no template) controls, and thus were not well suited for field application, so the use of those strips was discontinued and the PCRD cassettes were utilised. Another crucial modification during the optimisation was the addition of betaine into the RPA reaction mix, following determination of optimal concentration *via* titration. This reagent was used to prevent the formation of false-positive products generated in no-template controls due to secondary structure formation by the primers and probe.

### Assays performed at the field station

**Fig. 2:**
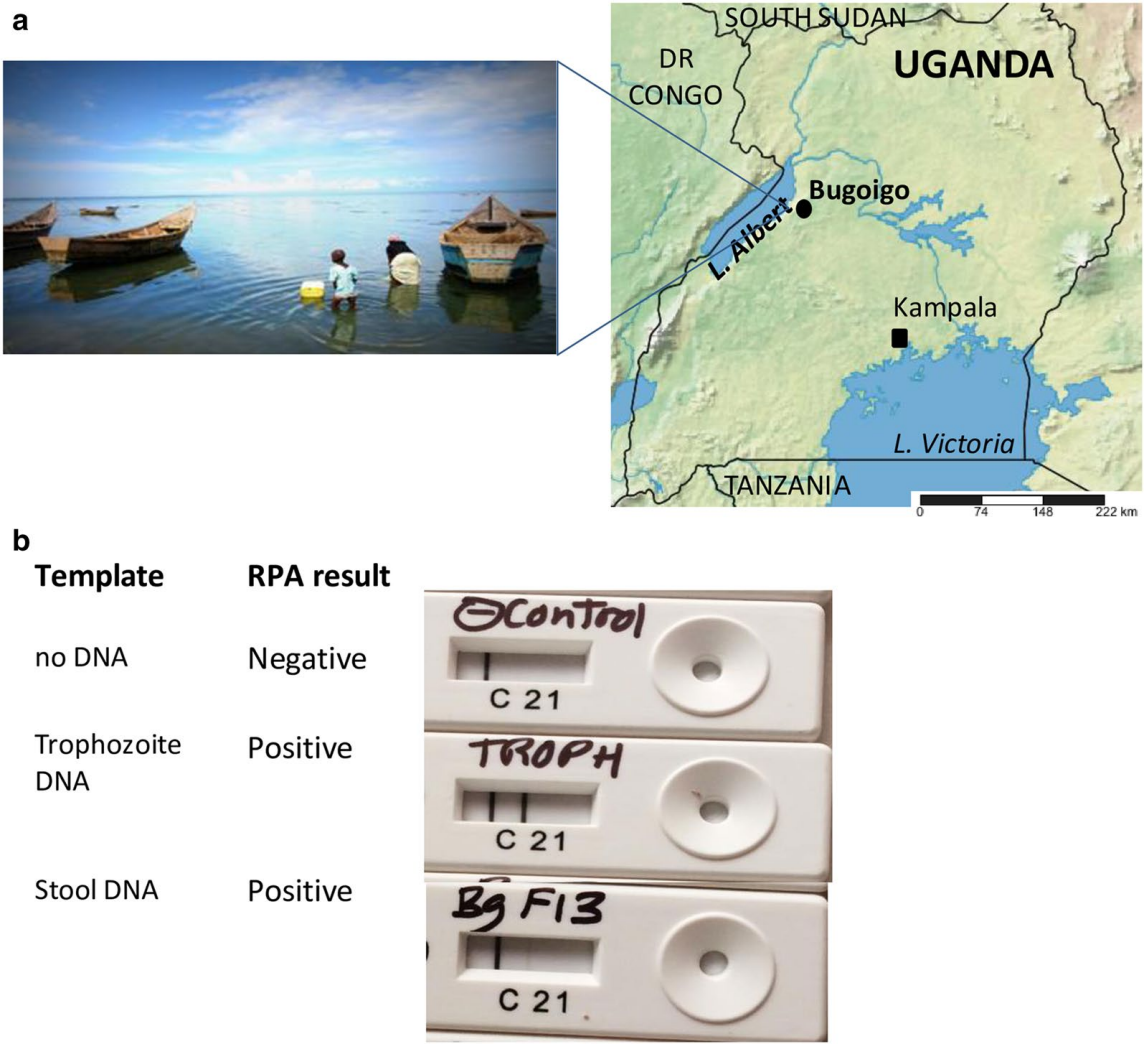
Lake Albert field setting. **a** Map of Uganda showing the location of the Bugoigo field site and lakeshore environment. **b** RPA under field conditions gave positive results for control DNA from *G. duodenalis* trophozoites and a stool sample. Photo credit: A. Bustinduy. Map source: http://www.simplemappr.net

The location and lakeshore environment of Bugoigo are shown Fig. 2a. The equipment used at the field station laboratory (including a microcentrifuge for spin-column DNA extraction and a dry heating block for isothermal incubation of RPA reactions) functioned reliably, allowing a total of 129 collected stool samples to be assayed. RPA gave positive results using control DNA from purified trophozoites, and also a single stool sample extracted in the field (Fig. 2b). However, the remaining samples extracted and tested in the field were negative by RPA, with no false-positives observed. Forty-nine stool samples from the 129 examined (37.9%) were positive using the *Giardia/Cryptosporidium* QuikChek (Table 1).

**Table 1:** Results of field samples assayed by QuikChek and *SSU* rDNA qPCR for *Giardia* (*n* = 129)

	Positive	Negative
QuikChek	49	80
*SSU* qPCR	30	99

### Subsequent assays performed at LSHTM on the field samples extracted using the QIAamp DNA Stool Mini Kit

Of the 129 samples tested 30 (23.25%) were positive for *G. duodenalis* by *SSU* rDNA qPCR (Table 1). Due to resource limitations, only a subset of 73 samples could be assayed by RPA at LSHTM. Of these, 21 (28.8%, 95% CI: 19.6–40.1%) were RPA-positive; 13 (17.8%, 95% CI: 10.6–28.3%) were positive by both RPA and the *SSU* rDNA qPCR (Table 2); and 10 (13.7%, 95% CI: 7.4–23.6%) were positive by all three assays, QuikChek coproantigen, *SSU* rDNA qPCR and RPA.

**Table 2:** Results comparing QuikChek and RPA with *SSU* rDNA qPCR (*n* = 73)

		*SSU* rDNA qPCR	
		Positive	Negative
QuikChek	Positive	18	10
	Negative	3	42
RPA	Positive	13	8
	Negative	8	44

**Fig. 3:**
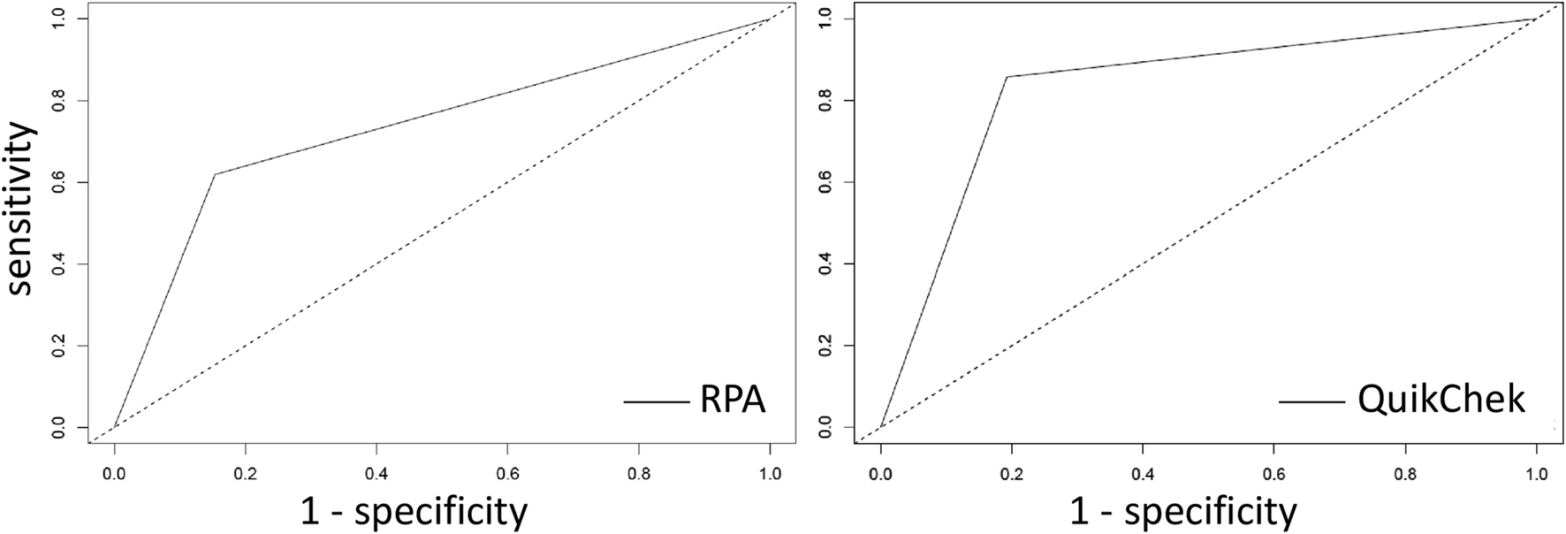
ROC curves of RPA and QuikChek against *SSU* rDNA qPCR

Within this subset, considering the *SSU* rDNA qPCR (*n* = 21 positive) as the ‘gold standard’, the QuikChek coproantigen test (18/21; 85.7%, 95% CI: 64.5–95.8%) was more sensitive than RPA (13/21, 61.9%, 95% CI: 40.1–79.3%), but with a similar specificity (80.8%, 95% CI: 67.9–89.4% *vs* 84.6%, 95% CI: 72.2–92.2%, respectively) (Table 2). Compared to the QuikChek coproantigen test, RPA had a sensitivity of 46.4% (95% CI: 29.5–64.2%) and a specificity of 82.2% (95% CI: 68.4–90.1%) (Table 3). Figure 3 shows the corresponding ROC curves for the gold standard *SSU* rDNA qPCR. For RPA, the area under the curve (AUC) was 0.73 (95% CI: 0.62–0.85) (*P* = 0.0001) and for QuikChek the AUC was 0.83 (95% CI: 0.74–0.93) (*P* < 0.0001).

**Table 3:** Results comparing QuikChek and RPA (*n* = 73)

		QuikChek	
		Positive	Negative
RPA	Positive	13	8
	Negative	15	37

For the *G*. *duodenalis* assemblage A and B qPCR, from the subset of 73 samples tested, assemblage typing was performed for 18 (24.7%) of the samples and characterised as: 9 samples as assemblage A; 5 samples as assemblage B; and 4 samples were of mixed assemblage infections for A and B.

## Discussion

In this study we found the RPA assay, when tested in the laboratory at the LSHTM, to have 61.9% sensitivity and 84.6% specificity, compared to the gold standard *SSU* rDNA qPCR for detecting *G. duodenalis* in faecal samples collected from school children in endemic villages around Lake Albert, Uganda. We have demonstrated here that the RPA assay can be used to amplify and detect specific DNA regions of *G. duodenalis* within faecal extracts, and that the assay is transposable to field station facilities, albeit with scope for further development, specifically in relation to the sample preparation. Crannell et al. [10] first developed the β-giardin RPA assay for *G. duodenalis*, which was assessed on spiked stool samples from healthy subjects and on samples from children in Peru, reporting 73% sensitivity against a gold standard of positivity by qPCR or microscopy, but acknowledged that the assay required further improvement and validation under field conditions.

Of note, during the course of this study, we encountered several unexpected technical issues, relating to faecal DNA extraction and subsequent RPA reaction and detection. Stool samples contain many inhibitors that are detrimental to molecular assays and it is crucial to reduce their presence as much as possible. Furthermore, faecal matter and *Giardia* cysts may require additional physical disruption to facilitate DNA extraction. The use of both the QIAamp DNA Mini Kit and QIAamp DNA Stool Mini Kit has been reported in the literature for stool *Giardia* DNA extraction. Initially during the RPA optimisation phase at LSHTM and at the Bugoigo field station, we used the QIAamp DNA Mini Kit in a modified protocol that included preliminary steps employing soil mix beads and NucliSENS lysis buffer prior to standard extraction protocol. For better field applicability, the possible effects on DNA extraction of local temperature and humidity should be considered. However, as an alternative method, we later changed to the QIAamp DNA Stool Mini Kit which greatly increased the performance of the RPA assay.

Additionally, the combination of betaine in the reaction mix and the use of the PCRD cassettes for detection [15], rather than the MGHD strips reported previously for this *Giardia* RPA [10], greatly increased the reliability of the RPA technique in our hands. We found that in the initial laboratory optimisation, the RPA assays without betaine were susceptible to giving false positives, although this could be controlled by using a concentration of 720 mM betaine with purified culture DNA; however, as this may have suppressed weak positives from stool-extracted DNA, we found after serial titration of spiked stool that this could be decreased to 70 mM for stool-extracted DNA. However, we cannot discount the possibility that the use of betaine may have also decreased the sensitivity of the assay. The AUC values from ROC curve analysis are encouraging, with scope for further optimisation. A future development of the RPA approach applied here to *Giardia* DNA would be the use of real time fluorescent RPA assays (TwistAmp exo; TwistDx). This uses a fluorescent probe that can be detected during DNA amplification and reactions can be run in small portable temperature-controlled fluorescence readers. These assays have also been reported to be more sensitive, have a reduced risk of cross-contamination and are more straightforward to carry out [16]. However, sample preparation is key for assay robustness and the need for the equipment needed for the extraction kits used reduces the field applicability of the RPA assay.

*Giardia duodenalis* subspecies assemblages A and B are considered to be human-specific [3]. Studies that have associated assemblage with clinical manifestations have not revealed a straightforward relationship, and there are conflicting reports from different settings of assemblages A or B being associated with diarrhoea and other symptoms [17]. The assemblage A- and B- specific qPCRs used here were first reported on giardiasis cases in the UK [11], with this same method later used on Ugandan [18] and Cambodian samples [19]. Assemblage typing from Ugandan human samples has reported assemblages A and B in single and mixed infections [18, 20–23]. Additional file 3: Figure S2 maps the assemblage typing from human samples from the present study and from the five previous reports, which have used the *tpi* gene and other genetic targets. As seen in the previous studies, we detected both assemblages A and B circulating in human populations in Uganda, and here in the Lake Albert environment. In addition to disease surveillance, this RPA assay may also be applicable to monitor therapeutic outcome of treatment and also to detection *Giardia* in faecal samples from infected animals.

### Study limitations

There are some limitations of this study. Issues relating to the extraction of high-purity DNA from stool samples impeded the field validations and should be further investigated, including alternative, more streamlined, methods for DNA extraction, such as that used for RPA with bovine stool samples [24], with emphasis on standardisation for field application. The absolute limit of detection of stool-extracted *Giardia* DNA by the RPA used in the present study should be fully determined, particularly in relation to that for *SSU* rDNA qPCR. Additionally, validation is needed on a set of well-defined clinical samples containing other gastro-intestinal pathogens to evaluate assay specificity, although the RPA-amplified sequence is specific to *G. duodenalis* by NCBI BLAST search.

## Conclusions

We have shown here that RPA can be applied successfully for the detection of *Giardia* DNA in field-collected stool samples for disease surveillance; however further optimisation of sample preparation, particularly with regard to DNA extraction from stool samples are required to make this RPA more suitable as point-of-care test, prior to subsequent field validations.

## Supplementary information

**Additional file 1: Table S1.** Sequences of primers and probes for the RPA and qPCR assays.

**Additional file 2: Figure S1.***Giardia duodenalis* qPCR targets. The figure shows the binding sites of primers (clear boxes) and probes (shaded boxes). Reverse primers are depicted on the corresponding sequence on the sense strand. **a** Assemblage specific qPCR of the *tpi* gene. The last either three or four bases at the 3’ end of the forward and reverse primers exploit assemblage A and B -specific polymorphisms of *tpi* and enable discrimination between assemblage A and B: assemblage A GDAT probe has 5’ VIC and 3’ BHQ1; assemblage B GDBT probe has 5’ FAM and 3’ NFQ. **b** Non-assemblage specific qPCR of the *SSU* rRNA gene: SSU probe has 5’ JOE and 3’ BHQ1. *Abbreviations*: FAM, JOE, VIC, fluorescent dyes; BHQ1, black hole quencher 1; NFQ, non-fluorescent quencher; nts, nucleotides; tpi, triose phosphate isomerase.

**Additional file 3: Figure S2.** Reports of *G. duodenalis* assemblage typing from Ugandan human samples. Numbers in brackets refer to cited reference list in main manuscript. *Abbreviation*: NP, National Park.

## Data Availability

The dataset supporting the conclusions of this article is included within the article and its additional files.
