# Supplementary material for: Application of a recombinase polymerase amplification (RPA) assay and pilot field testing for Giardia duodenalis at Lake Albert, Uganda
Source: Parasit Vectors. 2020 Jun 6;13:289. doi: 10.1186/s13071-020-04168-1 (PMC7275508; doi:10.1186/s13071-020-04168-1)
Supplement: Supplementary file 1 — Additional file 1: Table S1. Sequences of primers and probes for the RPA and qPCR assays. [file 13071_2020_4168_MOESM1_ESM.docx]

**Additional file 1: Table S1**. Sequences of primers and probes for the RPA and qPCR assays.

| **Reference** | **Assay (gene target, amplicon size)** | **Primer/Probe** | **Sequence 5' - 3'** |
| --- | --- | --- | --- |
| Crannell *et al.* [10] | RPA (β-giardin, 153 bp RPA product and 183 bp product ^a^) | RPA FOR | TAC GCT CAC CCA GAC GAT GGA CAA GCC CG |
|  |  | RPA REV ^b^ | **biotin**-TGT GCG ATG GCG TCC TTG ATC ATC TTC ACG C |
|  |  | RPA PROBE ^b^ | **FAM**-AGA CGG CGG TCA AGC TCA GCA ACA TGA ACC/**THF**/GCG CGT CAG CAG GTT - **block** |
| Elwin *et al****.*** [11] | qPCR assemblage A (*tpi*, 75 bp ^c^) | GDA FOR | CAT TGC CCC TTC CGC C |
|  |  | GDA REV | CTG CGC TGC TAT CCT CAA CTG |
|  |  | GDAT PROBE ^b^ | **VIC**-CCA TTG CGG CAA ACA-**MGB**-**NFQ** |
|  | qPCR assemblage B (*tpi*, 81 bp ^c^) | GDB FOR | GAT GAA CGC AAG GCC AAT AA |
|  |  | GDB REV | TCT TTG ATT CTC CAA TCT CCT TCT T |
|  |  | GDBT PROBE ^b^ | **FAM**-AAT ATT GCT CAG CTC GAG-**MGB**-**NFQ** |
| Verweij *et al****.*** [13, 14] | qPCR (ssu rRNA, 59 bp ^c^) | SSU FOR | GAC GGC TCA GGA CAA CGG TT |
|  |  | SSU REV | TTG CCA GCG GTG TCC G |
|  |  | SSU PROBE JOE ^b^ | **JOE**-CCC GCG GCG GTC CCT GCT AG-**BHQ1** |

Footnotes:

^a^ See Methods section of main manuscript text for derivation of the two amplicons

^b^ Non-nucleotide moieties are indicated in bold. BHQ1 = black hole quencher 1; Block = 3SpC3 moiety; MGB = minor groove binder; NFQ = non-fluorescent quencher; THF = tetrahydrofuran.

^c^ Refer to the cited references for derivations of limits of detection for these assays.
