# Supplementary figures and images for: Application of a recombinase polymerase amplification (RPA) assay and pilot field testing for Giardia duodenalis at Lake Albert, Uganda
Source: Parasit Vectors. 2020 Jun 6;13:289. doi: 10.1186/s13071-020-04168-1 (PMC7275508; doi:10.1186/s13071-020-04168-1)

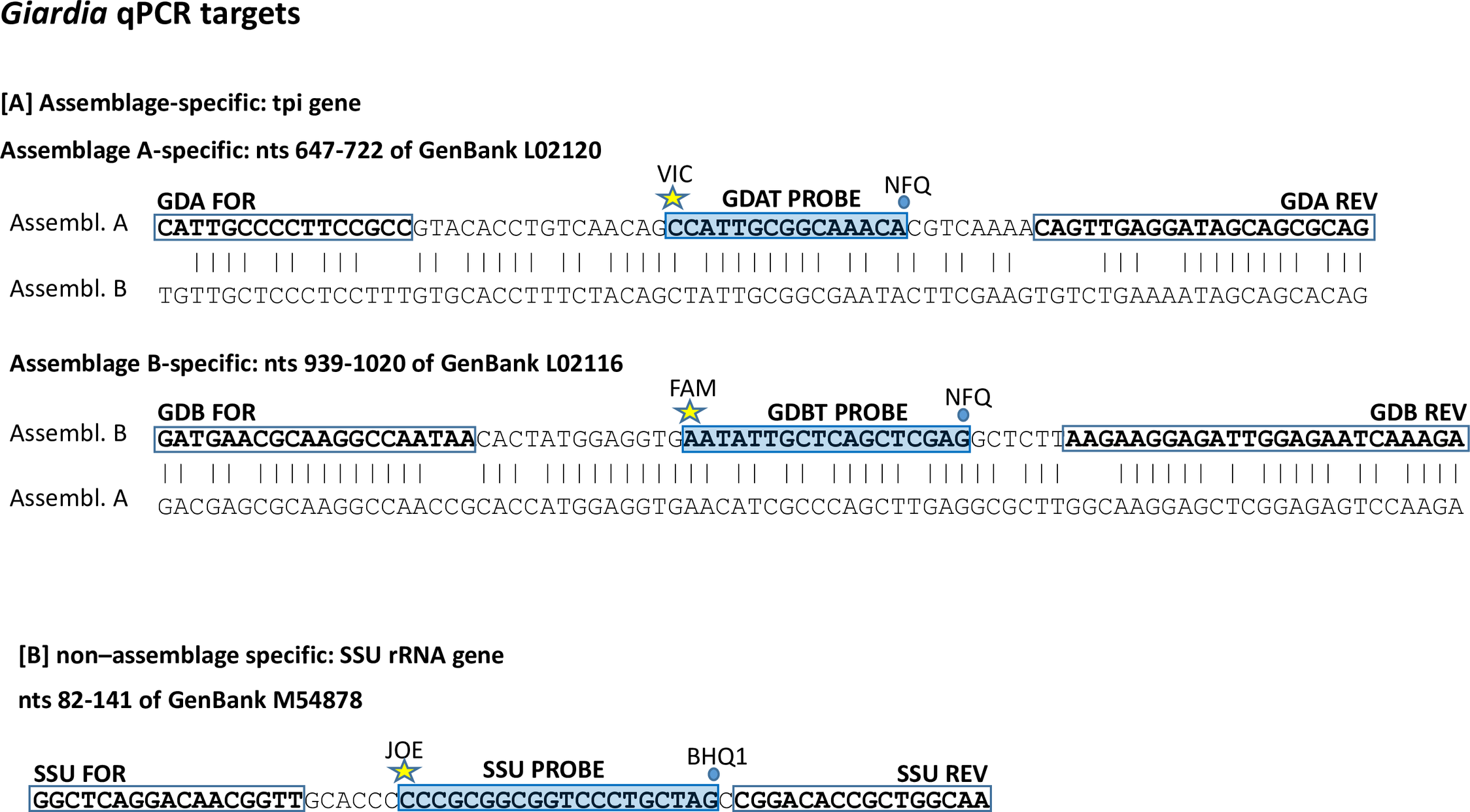

Supplement: Supplementary file 2 — Additional file 2: Figure S1.Giardia duodenalis qPCR targets. The figure shows the binding sites of primers (clear boxes) and probes (shaded boxes). Reverse primers are depicted on the corresponding sequence on the sense strand. a Assemblage specific qPCR of the tpi gene. The last either three or four bases at the 3’ end of the forward and reverse primers exploit assemblage A and B -specific polymorphisms of tpi and enable discrimination between assemblage A and B: assemblage A GDAT probe has 5’ VIC and 3’ BHQ1; assemblage B GDBT probe has 5’ FAM and 3’ NFQ. b Non-assemblage specific qPCR of the SSU rRNA gene: SSU probe has 5’ JOE and 3’ BHQ1. Abbreviations: FAM, JOE, VIC, fluorescent dyes; BHQ1, black hole quencher 1; NFQ, non-fluorescent quencher; nts, nucleotides; tpi, triose phosphate isomerase. [file 13071_2020_4168_MOESM2_ESM.tif]

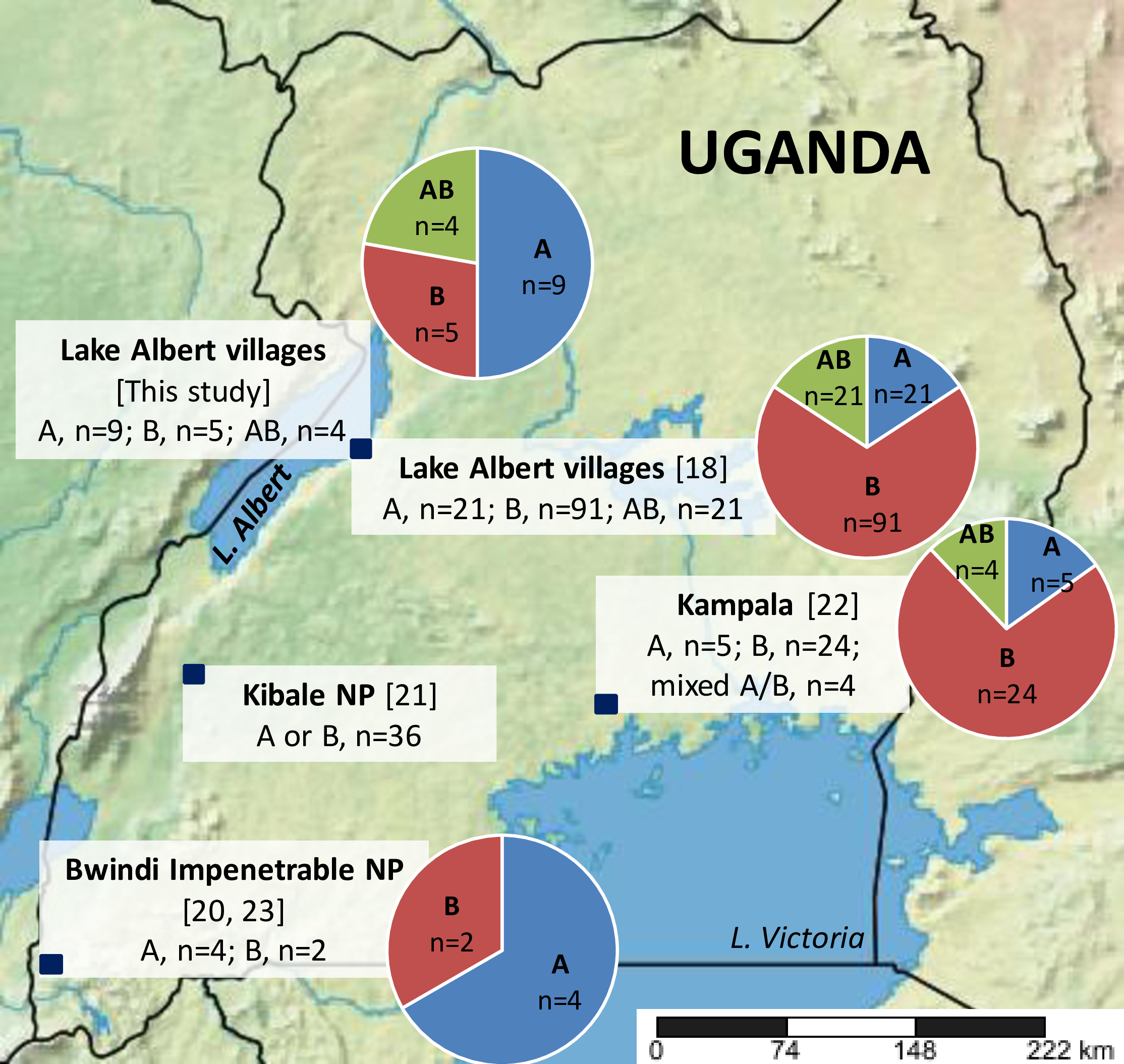

Supplement: Supplementary file 3 — Additional file 3: Figure S2. Reports of G. duodenalis assemblage typing from Ugandan human samples. Numbers in brackets refer to cited reference list in main manuscript. Abbreviation: NP, National Park. [file 13071_2020_4168_MOESM3_ESM.tif]
